# *APOE-ε4* Carrier Status and Gut Microbiota Dysbiosis in Patients With Alzheimer Disease

**DOI:** 10.3389/fnins.2021.619051

**Published:** 2021-02-24

**Authors:** Min Hou, Gaolian Xu, Maosheng Ran, Wei Luo, Hui Wang

**Affiliations:** ^1^School of Public Health, College of Medicine, Shanghai Jiao Tong University, Shanghai, China; ^2^Nano Biomedical Research Center, School of Biomedical Engineering, Shanghai Jiao Tong University, Shanghai, China; ^3^Department of Social Work and Social Administration, The University of Hong Kong, Hong Kong, China; ^4^Xinjin No. 2 People’s Hospital, Chengdu, China; ^5^Center for Single-Cell Omics, School of Public Health, Shanghai Jiao Tong University School of Medicine, Shanghai, China

**Keywords:** Alzheimer disease, apolipoprotein E, dysbiosis, genetic variants, gut microbiome

## Abstract

**Background:**

Alternations in gut microbiota and a number of genes have been implicated as risk factors for the development of Alzheimer disease (AD). However, the interactions between the altered bacteria and risk genetic variants remain unclear.

**Objective:**

We aimed to explore associations of the risk genetic variants with altered gut bacteria in the onset of AD.

**Methods:**

We collected baseline data and stool and blood samples from 30 AD patients and 47 healthy controls in a case-control study. The rs42358/rs4512 (*ApoE*), rs3851179 (*PICALM*), rs744373 (*BIN1*), rs9331888 (*CLU*), rs670139 (*MS4A4E*), rs3764650 (*ABCA7*), rs3865444 (*CD33*), rs9349407 (*CD2AP*), rs11771145 (*EPHA1*), and rs3818361/rs6656401 (*CR1*) were sequenced, and microbiota composition was characterized using 16S rRNA gene sequencing. The associations of the altered gut bacteria with the risk genetics were analyzed.

**Results:**

Apolipoprotein ε4 allele and rs744373 were risk loci for the AD among 12 genetic variants. Phylum Proteobacteria; orders Enterobacteriales, Deltaproteobacteria, and Desulfovibrionales; families Enterobacteriaceae and Desulfovibrionaceae; and genera *Escherichia–Shigella*, *Ruminococcaceae_UCG_002*, *Shuttleworthia*, *Anaerofustis*, *Morganelia*, *Finegoldia*, and *Anaerotruncus* were increased in AD subjects, whereas family Enterococcaceae and genera *Megamonas*, *Enterococcus*, and *Anaerostipes* were more abundant in controls (*P* < 0.05). Among the altered microbiota, APOE ε4 allele was positively associated with pathogens: Proteobacteria.

**Conclusion:**

The interaction of APOE ε4 gene and the AD-promoting pathogens might be an important factor requiring for the promotion of AD. Targeting to microbiota might be an effective therapeutic strategy for AD susceptible to APOE ε4 allele. This needs further investigation.

## Introduction

Alzheimer disease (AD) is the most common form of dementia (Masters et al., 2015). More than 47 million people are currently afflicted worldwide, with this number predicted to reach 131.5 million by the year 2050 (Bahar-Fuchs et al., 2019). By 2010, 5.69 million Chinese people were living with AD, a 3-fold increase from the previous decade (Clay et al., 2019), which have been partially attributed to an increasing elderly population. Symptoms include a progressive and global deterioration in memory, learning, orientation, language, comprehension, and judgment (Nussbaum and Ellis, 2003). The main pathology of AD involves a higher level of extracellular amyloid-beta (Aβ) peptide in brain tissue, depositing in diffuse and neuritic plaques, and intracellular hyperphosphorylated tau (a microtubule assembly protein, p-tau) accumulating as neurofibrillary tangles (Dubois et al., 2016). However, pathogenesis has not been fully elucidated. Moreover, no treatments or interventions have been found to date that can effectively mitigate the progression of AD.

Risk factors for the development of AD have been identified in previous publications and include a genetic predisposition (Moustafa et al., 2018) and environmental factors (Daviglus et al., 2010; Barnes and Yaffe, 2011). Genome-wide association studies have identified polymorphisms with various levels of risk to develop AD, such as *ABCA7*, *BIN1*, *CASS4*, *CD33*, *CD2AP*, *CELF1*, *CLU*, *CR1*, *DSG2*, *EPHA1*, *FERMT2*, *HLA-DRB5-DBR1*, *INPP5D*, *MS4A*, *MEF2C*, *NME8*, *PICALM*, *PTK2B*, *SLC24H4-RIN3*, *SORL1*, and *ZCWPW1* (Harold et al., 2009; Lambert et al., 2009; Moustafa et al., 2018). Particularly, the greatest genetic risk factor is apolipoprotein E (APOE) genotype, with the presence of a single ε4 allele increasing the risk by 3- to 4-fold compared with ε2 or ε3 allele (Corder et al., 1993). The APOE functions to transport cholesterol and other lipids to cells, facilitate their cellular uptake (Mahley, 1988), and to promote Aβ clearance and neuronal signaling (Herz and Beffert, 2000). However, the specific functions of APOE that are associated with the development of AD remain unclear.

Recent evidence also implicates a role of the gut microbiome in the development of AD (Bonfili et al., 2017; Vogt et al., 2017; Zhang et al., 2017; Zhuang et al., 2018; Liu et al., 2019; Saji et al., 2019; Kim et al., 2020). The human gut microbiome consists of approximately 10^14^ microbes, 10 times the number of cells present in host, and is dominated by Firmicutes (60–80%) and Bacteroidetes (20–30%) species (Ley et al., 2006; Consortium, 2012). The gut microbiome in patients with AD has been identified as distinct in composition compared with subjects without cognitive impairment. For example, Bifidobacteria are reduced (Vogt et al., 2017), whereas Lachnospiraceae (Vogt et al., 2017), Gammaproteobacteria, Enterobacteriales, and Enterobacteriaceae species have been shown to be increased (Liu et al., 2019).

It has been suggested that the gut microbiome regulates multiple neurochemical pathways (Bonfili et al., 2017; Sun et al., 2020). The gram-positive bacteria *Lactobacillus brevis* and *Bifidobacterium dentium* enable the production of γ-aminobutyric acid (GABA), a major inhibitory neurotransmitter in human central nervous system (CNS) (Barrett et al., 2012), and lower concentrations of GABA have been found in the frontal, temporal, and parietal cortex of patients with AD compared with individuals without AD (Lanctot et al., 2004). Inversely, the neurotoxins saxitoxin and anatoxin-α, produced by various *Cyanobacteria*, have been shown to contribute to AD progression (Brenner, 2013). Moreover, gut dysbiosis may increase the levels of undesirable microbial metabolites in brain tissues such as lipopolysaccharides as a result of increased permeability of both the intestinal and the blood–brain barrier (Quigley, 2017). Current knowledge asserts that multiple factors shape the gut microbiome, including age, genetics, and the diet of the host (Benson et al., 2010). However, few studies have investigated factors impacting gut bacteria that were different between individuals with AD and without AD.

Several recent studies have demonstrated that specific genetic loci contribute to alterations in gut microbial composition, such as HLA genes for autoimmunity (Russell et al., 2019), *NOD2* for inflammatory bowel disease (Aschard et al., 2019), and *MUC19* for primary sclerosing cholangitis (Eksteen, 2014). A recent study also evaluated associations between APOE genotypes and the abundance *of Prevotellaceae*, *Ruminococcaceae*, and several butyrate-producing species in healthy humans aged between 56 and 78 years (Tran et al., 2019). However, this has not yet been investigated in other populations, especially in those with AD. Thus, the extent to which human genetics related to AD that shape microbiome composition, especially specific gut microbes for AD, remains unclear. Thus, in this study, we explored genetic factors to AD and gut bacteria that were different between patients with AD compared with cognitive healthy people, as well as the effects of identified genetic variants on the bacteria.

## Materials and Methods

### Study Population

Participants were recruited from Xinjin, Chengdu, China. Two hundred fifty-two subjects were diagnosed with dementia due to AD, after retrieving the medical records from community hospitals and an epidemiological survey on mental health by a multidisciplinary team of neurologists, neuropsychologists, sociologists, and nurses in 2015. The diagnosis of AD accorded with the *Diagnostic and Statistical Manual of Mental Disorders, Third Edition*, revised, dementia criteria; the National Institute of Neurological and Communicative Disorders and Stroke; and the Alzheimer Disease and Related Disorders Association criteria, including both possible and probable AD (McKhann et al., 1984). In the end, 105 patients with AD and 554 healthy participants completed questionnaires by caregivers or themselves in 2018. Since then, these participants will be seen every 3 years, and annually for 80 years. These questionnaires were repeated in the follow-up inquiries.

As the effects of age and gender on AD incidence are well-documented (Fratiglioni et al., 1991; Vermunt et al., 2019), these factors were used as matching variables in this study. Controls were matched on age (within 5 years) and sex. If we were not able to find suitable controls for all AD cases using the matching criteria, two more matching cycles were performed. Criteria were relaxed in each cycle to obtain more matches. If there were not enough controls, stratified random sampling based on age and sex was used to maintain overall similarities between AD patients and controls. There were 30 patients with AD included in the final study aged 60–80 years, and there were 47 subjects without cognitive impairment; their gut microbiota speciation and polymorphisms were analyzed. Individuals included as controls underwent an assessment and were examined as having normal cognition. A screening questionnaire was provided to subjects for exclusion including history of diseases, use of medicine, antibiotics, nutritional supplements, and questions related to diet. Exclusion criteria for this study included any significant neurologic disease, gastrointestinal disease, chronic constipation, *Clostridium difficile* infection, history of alcohol/substance dependence, major psychiatric disorders, or any cancers. Those who have received antibiotics for at least 3 months prior to sampling, have any eating disorder; have dietary change for at least 1 month; were on any nutritional supplement such as probiotics or special diet; and have history of gastrointestinal operations that could confound with the results of gut microbiota were excluded for fecal collection. All participants or family caregivers provided written informed consent before involvement in this study. The Ethics Committee of School of Public Health at Shanghai Jiao Tong University for Human Subject Research approved all study procedures, and all experiments were performed in accordance with relevant guidelines and regulations.

### Clinical Information

On the morning of blood sample collection, pulse and blood pressure were measured using a validated digital electronic device (HEM-7080IC; Omron Healthcare, Kyoto, Japan) by public health nurses. Measurement of fasting glucose, serum creatinine, total cholesterol, high-density lipoprotein (HDL) cholesterol, low-density lipoprotein (LDL) cholesterol, and triglycerides were obtained from the latest clinical records within 1 year.

### Stool Sample Collection and Fecal DNA Extraction

Fecal samples were obtained from participants by family caregivers or themselves at home and immediately returned by delivery sample collection kits, packaged within insulated containers, and chilled with frozen gel packs for transportations to community hospitals. Finally, fecal samples were successfully collected from 21 patients with AD and 40 controls. All fecal samples were processed on the day of fecal collections. Upon receipt, chilled samples were weighed, and 200 mg of aliquots was prepared into sterile bead beating tubes and remained frozen at −80°C until DNA isolation. Full details of the DNA extraction are in online Supplementary Material.

### V3-V4 16S Sequencing Using Illumina MiSeq 2 × 300 bp

Illumina MiSeq system was used to generate nucleotide-sequencing data for 61 samples with 436 of sequencing average length. Sequencing read preprocessing, including merging, and demultiplexing, was done. Additional details are shown in the Supplementary Material.

### Operational Taxonomic Units Picking and Filtering

The sequences were then clustered into operational taxonomic units (OTUs) using UPARSE (version 7.1)^1^ with a novel “greedy” algorithm with 97% similarity and taxonomically classified using the Greengenes 13.5 reference database (McDonald et al., 2012). Additional details are in the Supplementary Material.

### Single-Nucleotide Polymorphism Selection and Genotyping

Blood (6 ml) was obtained from participants in the morning using EDTA blood-collecting tube in each community hospitals after the 12 h fasting. The 0.5 ml of whole blood was separated in the cold and frozen at -80°C until DNA extraction. DNA was extracted from whole blood with the use of the Qiagen DNA blood kit (Qiagen, Germantown, MD, United States). The remaining blood sample was separated from by centrifugation at 3000 × *g* for 15 min at 4°C, and plasma was collected and stored at −80°C for other analysis.

### DNA Purification and Quality Control Procedures

Total twelve reported AD genetic risk variants were selected for the single-nucleotide polymorphism (SNP)–microbiota interaction analyses including rs42358 (*ApoE*), rs4512 (*ApoE*), rs3851179 (*PICALM*), rs744373 (*BIN1*), rs9331888 (*CLU*), rs670139 (*MS4A4E*), rs3764650 (*ABCA7*), rs3865444 (*CD33*), rs9349407 (*CD2AP*), and rs11771145 (*EPHA1*) and genetic variants in *CR1*: rs3818361 and rs6656401. We selected these risk variants ensuring that the selected genetic risk SNPs are functional variants or are in strong linkage disequilibrium with functional variants (Bradshaw et al., 2013; Imhann et al., 2018) to explore the interaction of the host with the gut microbiota. The DNA fragments containing the SNP sites including rs3851179 (*PICALM*), rs11771145 (*EPHA1*), rs3818361 (*CR1*), rs6656401 (*CR1*), rs7412 (*APOE*), and rs429358 (*APOE*) were amplified by polymerase chain reaction (PCR) with specific primers (Supplementary Table 1). The PCR products of these fragments were directly sequenced using the PCR amplification primers (Germer et al., 2000) with the data shown in online Supplementary Figure 1. Other AD genetic risk variants included rs744373 (BIN1), rs9331888 (CLU), rs670139 (MS4A4E), rs3764650 (ABCA7), rs3865444 (CD33), and rs9349407 (CD2AP), with the genotype tested using with allele-specific TaqMan assays in LightCycler^®^ 480 Instrument II (Roche Life Science) with 96-Well Block Module. The PCR primers and TaqMan probes are listed in online Supplementary Table 1. The genotype data were analyzed using the LightCycler software version 1.1. The APOE haplotypes (ε2/ε3, ε3/ε3, ε3/ε4, and ε4/ε4) were derived from the allelic combinations of the APOE SNPs rs7412 and rs429358.

### Statistical Analysis

Sample size for this study was determined using QUANTO version 1.2.4^2^. As the importantly strongest genetic risk factor for AD, assuming minor allele frequencies (MAFs) from 0.1, this study sample size of 77 cases is suitable to detect an allelic odds ratio (OR) of 4.5 at 80% statistical power and 5% significance level.

The continuous variables of baseline characteristics presented in this study were expressed as the means ± SD or medians with interquartile range (IQR). And the categorical variables were expressed as frequencies and percentages. The normal distribution of variables including age, body mass index (BMI), waist, pulse, systolic blood pressure, diastolic blood pressure, fasting glucose, serum creatinine, total cholesterol, HDL cholesterol, LDL cholesterol, and triglycerides was assessed using the Shapiro–Wilk test. Baseline characteristics between patients with AD and controls were compared, using χ^2^, Fisher exact test, Wilcoxon–Mann–Whitney test, or Student *t*-test, where appropriate. *P* < 0.05 was considered statistically significant. Analyses were completed using the IBM SPSS program, version 22 (IBM, Chicago, IL, United States).

### SNPs and APOE Genotype Analysis

Analyses of genetic sequencing data were completed using the IBM SPSS program mentioned previously. SNPs with Hardy–Weinberg equilibrium test *p* < 0.05 or MAF < 1% were excluded. A χ^2^ test was used to estimate the differences between the frequencies of the genotypes and alleles in patients with AD and the control group. Differences were considered significant at *P* < 0.05.

Because certain APOE genotypes are relatively rare (e.g., ε2/ε2 and ε4/ε4), the APOE genotype ORs were calculated by dividing subjects into two main categories: those with at least one ε4 allele present and those with no ε4 allele. The associations between the candidate SNPs, APOE ε4 allele and AD were estimated using ORs with 95% confidence intervals in logistic regression models, which were adjusted by including significant covariates, mainly age, gender, and APOE genotype.

### Microbial Data Processing

The sequence data of the gut microbiota was mainly analyzed using the QIIME (1.9.1) (Caporaso et al., 2010) and R packages (v3.2.0). Beta diversity distances were calculated using Bray–Curtis dissimilarity and weighted/unweighted-UniFrac represented in principal coordinate analyses (PCoA) at OTU level. To detect statistical differences in beta diversity metrics between the AD and control groups, the permutational multivariate analysis of variance in the *vegan* package was used. Statistically significant differences in the relative abundances of taxa from phylum to species were calculated by using the linear discriminant analysis effect size method (LEfSe) with nonparametric factorial Kruskal–Wallis rank-sum test. Taxa with linear discriminant analysis (LDA) results of more than three were considered statistically significantly enriched.

### Quantitative Trait Locus Association Analysis

To link microbial composition to genetic variation, abundance values of taxonomies were treated as quantitative traits. Multivariate analysis was performed using multivariate association with linear models (MaAsLin) (Morgan et al., 2012) to identify associations of specific gut microbial taxa at all taxonomic levels from kingdom to genus with AD-related SNPs and genotypes. Associations with a Benjamini and Hochberg false discovery rate (FDR)–corrected *p*-value (*q*-value) of <0.05 were considered to be significant. Gut microbiota differences among subgroups defined by AD were assessed by the Wilcoxon–Mann–Whitney tests.

## Results

### Characteristics of Patients With AD and Cognitive Healthy Controls

As shown in Table 1, the mean subject ages were, respectively (SD = 71.9 ± 6.9) years and (SD = 71.1 ± 6.7) years. Meanwhile, 43.3% and 53.2% of the subjects were female in the AD group and control group, respectively, but not significantly different between groups (*p* > 0.05). In addition, the anthropometric characteristics including BMI, waist circumstance, pulse and blood pressure, and sociodemographic factors including educational level and marriage status were not significantly different between groups (*p* > 0.05). Moreover, the biochemical analyses including fasting glucose, total cholesterol, HDL cholesterol, LDL cholesterol, and triglycerides were not significantly different between groups (*p* > 0.05). There were 23.3% of AD patients with type 2 diabetes, but no significant difference compared with the control group (*p* > 0.05). The 43.3% of patients with AD had hypertension, and 13.3% of those had cardiovascular disease, and these numbers were significant different as compared to the healthy control group (*p* = 0.032; *p* = 0.011).

**TABLE 1:** Participant characteristics in this study (*n* = 77).

	Patients with AD (*n* = 30)	Controls (*n* = 47)	*p*-value
Age (years)	71.9 ± 6.9	71.1 ± 6.7	0.966
**Gender**			
Male	17 (56.7)	22 (46.8)	0.402
Female	13 (43.3)	25 (53.2)	
BMI (kg/m^2^)	23.74 ± 4.77	23.63 ± 3.31	0.994
Waist (cm)	77.47 ± 8.92	79.33 ± 9.06	0.935
**Medical history**			
Hypertension	13 (43.3)	22 (50)	0.032
Type 2 diabetes	7 (23.3)	3 (6.8)	0.767
Cardiovascular disease	4 (13.3)	0 (0)	0.011
**Marriage**			0.321
Single	0 (0)	3 (6.8)	
Married	30 (100.00)	44 (93.2)	
**Education**			0.527
No	8 (26.7)	9 (19.1)	
Primary school	13 (43.3)	26 (55.3)	
Secondary high school and higher level	9 (30.0)	12 (25.6)	
Pulse	75.00 ± 12.42	73.37 ± 9.77	0.440
Systolic blood pressure (mm Hg)	131.84 ± 14.59	138.61 ± 16.18	0.436
Diastolic blood pressure (mm Hg)	79.74 ± 9.76	80.63 ± 9.64	0.803
Fasting glucose (mmol/L)	6.12 ± 1.27	5.47 ± 1.06	0.193
Serum creatinine (μmol/L)	71.51 ± 37.31	67.13 ± 28.06	0.165
Total cholesterol (mmol/L)	4.56 ± 0.90	4.99 ± 0.76	0.310
HDL cholesterol (mmol/L)	1.30 ± 0.34	1.46 ± 0.67	0.376
LDL cholesterol (mmol/L)	2.57 ± 0.71	2.73 ± 0.69	0.603
Triglycerides (mmol/L)	1.28 ± 0.53	1.49 ± 0.68	0.384

*Data are n(%), mean ± SD, or median (IQR), unless otherwise specified.*

### Genetic Variants and AD

Using the χ^2^ test, the significant associations of AD status were found with APOE genotype and MS4A4E (rs670139), but not with PICALM (rs3851179), EPHA1 (rs11771145), CR1 (rs3818361), CR1 (rs6656401), CD33 (rs3865444), ABCA7 (rs3764650), CLU (rs9331888), BIN1 (rs744373), or CD2AP (rs9349407) (Table 2). Fifteen participants were APOE ε4 carriers (APOE ε3/ε4 and APOE ε4/ε4; 12 men and 12 women), among whom 10 were patients with AD. As expected, the frequency of APOE ε4 carriers was significantly greater in patients with AD compared with normal cognitive individuals. The presence of at least one APOE ε4 allele significantly increased the risk of AD compared with the absence of APOE ε4 allele (*p* = 0.043; OR, 4.38; 95% CI, 1.05–18.30). In addition, harboring a C allele in rs7744373 (BIN1) was associated with greater risk of AD compared with the homozygous TT genotype after adjustment for age and gender (*p* = 0.009; OR, 6.63; 95% CI, 1.57–25.41).

**TABLE 2:** The association of genetic variants with AD risk.

	General genetic model^*a*^				Crude genetic model^*b*^		Adjusted genetic model^*c*^	
		
	Genotype frequencies (%)				OR (95% CI)	*p*-value	OR (95% CI)	*p*-value
			
*ApoE*	–/–	ε3/ε4	ε4/ε4	*p*-value	(ε4/ε4) + (ε3/ε4) vs. –/–			
Control subjects	42	5	0	**0.022**	4.38	**0.043**	–	–
AD patients	20	9	1		(1.05–18.30)		–	
rs3851179 (*PICALM*)	GG	AG	AA		AG+GG vs. AA			
Control subjects	13	27	7	0.795	1.47	0.589	1.48	0.587
AD patients	9	17	4		(0.36–5.97)		(0.36–6.11)	
rs11771145 (*EPHA1*)	AA	AG	GG		AG+AA vs. GG			
Control subjects	19	20	8	0.669	1.86	0.311	1.69	0.405
AD patients	8	19	3		(0.56–6.19)		(0.49–5.77)	
rs3818361 (*CR1*)	CC	CT	TT		CT+TT vs. CC			
Control subjects	20	20	7	0.289	2.77	0.123	2.71	0.133
AD patients	9	15	6		(0.76–10.11)		(0.74–9.98)	
rs6656401 (*CR1*)	GG	GA	AA		GA+AA vs. GG			
Control subjects	18	29	0	0.075	0.43	0.142	0.43	0.145
AD patients	19	11	0		(0.14–1.33)		(0.14–1.35)	
rs3865444 (*CD33*)	GG	GT	TT		GT+TT vs. GG			
Control subjects	31	15	1	0.290	0.27	0.086	0.29	0.111
AD patients	24	5	1		(0.06–1.20)		(0.06–1.33)	
rs3764650 (*ABCA7*)	TT	GT	GG		GT+GG vs. TT			
Control subjects	23	21	0	0.929	2.06	0.260	1.98	0.297
AD patients	23	21	1		(0.59–7.29)		(0.55–7.12)	
rs9331888 (*CLU*)	CC	CG	GG		CG+GG vs. CC			
Control subjects	10	17	20	0.401	1.91	0.414	1.78	0.474
AD patients	6	16	8		(0.40–9.01)		(0.37–8.70)	
rs744373 (*BIN1*)	TT	CT	CC		CT+CC vs. TT			
Control subjects	23	20	4	0.251	6.32	**0.009**	6.63	**0.009**
AD patients	8	20	1		(1.57–25.41)		(1.60–27.49)	
rs670139 (*MS4A4E*)	CC	AC	AA		CT+TT vs. CC			
Control subjects	11	21	15	**0.015**	0.37	0.136	0.36	0.120
AD patients	13	14	3		(0.10–1.37)		(0.10–1.31)	
rs9349407 (*CD2AP*)	GG	CG	CC		CG+CC vs. GG			
Control subjects	40	7	0	0.835	0.48	0.44	0.47	0.424
AD patients	25	5	0		(0.08–3.05)		(0.07–3.04)	

*P < 0.05 are in bold type.^a^χ^2^ test.^b^Adjusted for age and gender.^c^Adjusted for age, gender and APOE.CI, confidence interval; OR, odds ratio; –/–, non-ε3 or -ε4.*

### Subjects With AD Harbor the Altered Gut Microbiota

**FIGURE 1:**
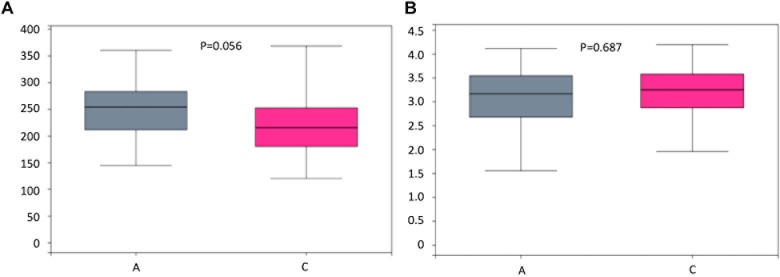
Boxplots and statistical comparison (Wilcoxon rank-sum test) shows no significant difference between alpha diversities among groups including Sobs index **(A)** and Shannon index **(B)**. *P*-values for each comparison are depicted above the boxplots of the groups being compared. Boxplot medians (center lines); interquartile ranges (box ranges). Patients with AD and healthy subjects are, respectively, colored in gray and rose red.

**FIGURE 2:**
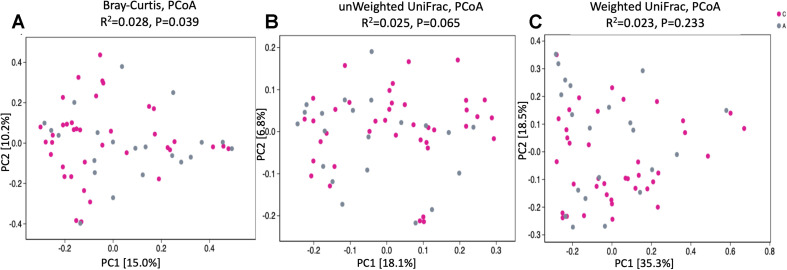
PCoA of bacterial beta diversity based on **(A)** Bray–Curtis dissimilarity, **(B)** Unweighted, and **(C)** Weighted UniFrac distances. Patients with Alzheimer disease and healthy subjects are, respectively, colored in gray and rose red and are indicated by circles.

To assess bacterial composition, we successfully collected 60 fecal samples, among which 21 subjects were patients with AD and 40 were cognitively healthy controls. In total, more than 4.5 million quality-filtered sequences were obtained (49,274 ± 11,495 sequences per sample). We identified a total of 808 bacterial taxa across six hierarchical levels from phylum to species at 12,556 OTUs. The predominant phyla in both patients with AD and cognitive healthy controls were Firmicutes (52% in patients with AD, 59% in cognitive healthy controls), Proteobacteria (24% in patients with AD, 12% in cognitive healthy controls), and Bacteroidetes (18% in patients with AD, 22% in cognitive healthy controls). Clostridia was the most abundant class in patients with AD (42%) and in healthy controls (49%). *Escherichia–Shigella* was the most abundant genera in patients with AD (19%), whereas *Bacteroides* was the most abundant genus in healthy controls (12%). Analysis of alpha diversity revealed no significant differences between AD and cognitive healthy subjects in the Sobs index (*p* = 0.056) and Shannon index (*p* = 0.687) using Wilcoxon rank-sum test as depicted in Figure 1. To test significant differences in the structure of gut microbiota responding to AD status, we next performed analysis of similarities on the β-diversity distances with unweighted/weighted UniFrac and Bray–Curtis distances. The PCoA analysis presented slight difference in gut microbial composition between groups calculated on the Bray–Curtis dissimilarity (*p* = 0.039), but hardly revealed distinguishable bacterial microbiota of AD subjects compared to cognitive healthy controls on the unweighted UniFrac (*p* = 0.065) and the weighted UniFrac (*p* = 0.233) (Figure 2).

**FIGURE 3:**
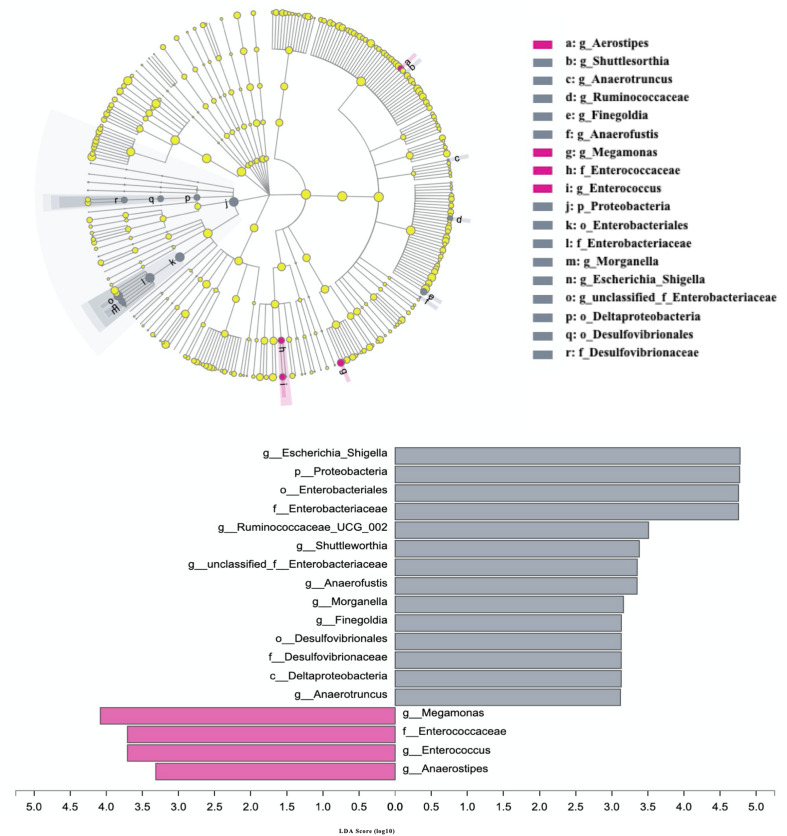
Differences in the composition of gut microbiome between patients with AD and cognitive healthy subjects. Patients with Alzheimer disease and healthy subjects are, respectively, colored in gray and rose red. Gray bars and circles indicate significant.

The differences in the abundances of gut microbial taxa between AD patients and cognitive healthy subjects were analyzed using LEfSe with nonparametric factorial Kruskal–Wallis rank-sum test with the filtered set excluding low abundance (<0.1% mean relative abundance) and <3. The results indicated 18 taxa were significantly altered in patients with AD from phylum to genera (*p* < 0.05) (Figure 3). Among those excluding unclassified, uncultured, and non-rank taxa, phylum Proteobacteria; orders Enterobacteriales, Deltaproteobacteria, and Desulfovibrionales; families Enterobacteriaceae and Desulfovibrionaceae; genera *Escherichia–Shigella*, *Ruminococcaceae_UCG_002*, *Shuttleworthia*, *Anaerofustis*, *Morganelia*, *Finegoldia*, and *Anaerotruncus* were increased in AD subjects, whereas family Enterococcaceae and genera *Megamonas*, *Enterococcus*, and *Anaerostipes* were more abundant in healthy controls (*P* < 0.05).

### Associations of Risk Genetic Variants With the Altered Gut Microbiota

**FIGURE 4:**
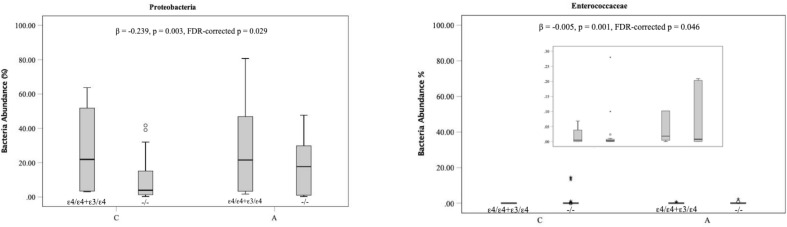
Associations of AD associated genetic variants with specific members of the gut microbiota. The boxplots indicate the median (horizontal solid line) and the IQR between the first and third quartiles (box). ^∗^indicate significance FDR < 0.05. A, Alzheimer disease; C, Cognitive healthy subjects.

To investigate associations between two AD risk genetic variants in the gene *BIN1* (rs744373) and *APOE* and specific microbial taxa related to AD, the targeted analyses were performed using MaAsLin. Two significant associations were detected between APOE genotypes and AD-related gut microbial taxa. A higher number of the ε4 allele was associated with an increase in the abundances of the phylum Proteobacteria (FDR-*p* = 0.029) and the family Enterococcaceae classified into the phylum Firmicutes (FDR-*p* = 0.046) as depicted in Figure 4 (FDR < 0.05). The β-coefficients suggest that the associations of the APOE genotype with the phylum Firmicutes are of moderate effect size (β = −0.23). In particular, bacteria displaying higher levels in carriers of AD genetic risk alleles are more likely to be positively associated with the risk of AD. In addition, each additional copy of the minor A allele at non–AD-associated gene *PICALM* SNP rs3851179 increased the abundances of the Actinobacteria phylum with a light effect size (β = −0.08, FDR = 0.034).

## Discussion

In this study, we explored genetic risks associated with AD and report that genetic variants including APOE genotype and *BIN1* (rs744373) are significantly associated with abundance of specific gut microbiota. In particular, we found that the phylum Proteobacteria and the family Enterococcaceae are strongly associated with AD status, and their abundance is significantly increased in those subjects with an *APOE* ε4 genotype, leading to an increased risk of AD.

We found that both *APOE* ε4 genotype and an SNP in rs7744373 (*BIN1*) gene were significant risks for AD in a population after adjustment for age and gender, which is consistent with previous studies in Chinese populations (Tan et al., 2013; Han et al., 2019) and European–American subjects (Wijsman et al., 2011). After adjusting for *APOE* ε4 genotype, the positive association with AD by C allele in rs7744373 (*BIN1*) remained. Previous studies indicate that *BIN1* is expressed to regulate synaptic vesicle endocytosis and cytoskeletal dynamics (Cousin and Robinson, 2001), and its isoforms were different in multiple AD brain regions (Holler et al., 2014). It may act as a modulator of tangle pathology (Holler et al., 2014), but the exact function of this SNP still remains unknown.

Although a significant association with AD was not found in rs670139 (MS4A4E), the *p*-value for frequencies of the rs670139 alleles (CC:AC:AA) was significant (*p* = 0.015) in our analysis. Hence, further assessment of this SNP in a large sample with sufficient statistical power is needed.

The results indicated that 18 taxa were significantly altered in patients with AD from phylum to genera when compared to non-AD individuals (*p* < 0.05) (Figure 3). Decreased gut microbiota diversity has been reported in subjects with AD (Vogt et al., 2017; Liu et al., 2019). In this study, these 18 bacterial taxa characterized dysbiosis in the fecal microbiota of patients with AD. The expansion of the phylum Proteobacteria in the microbial profiles of patients with AD was in agreement with recent findings of Liu et al. (2019) in patients with AD from a hospital in Hangzhou, East China. In addition, in this study, the increase of Enterobacteriaceae, a member of Proteobacteria, has been reported previously in patients with other CNS diseases, such as Parkinson disease (Unger et al., 2016) and major depressive disorder (Jiang et al., 2015). Interestingly, species *Escherichia–Shigella* and *Morganelia* as important pathogens belonging to Enterobacteriaceae (data not shown) were also enriched in patients with AD compared to healthy individuals and are catalase-positive and oxidative-negative *in vitro* (Adeolu et al., 2016). Importantly, we newly identified the reduction of genera *Megamonas*, *Enterococcus*, and *Anaerostipes* in patients with AD. Megamonas has been shown to produce short-chain fatty acids (SCFAs) derived from dietary fibers (Adeolu et al., 2016), which may benefit host energy metabolism to attenuate the development of AD (Ho et al., 2018). In addition, *Anaerostipes*, a member of family Lachnospiraceae, are also SCFA-producing bacteria (Schwiertz et al., 2002) and were detected at higher levels in non-AD Chinese rather than non-AD Caucasian as compared to AD subjects in previous works (Zhuang et al., 2018; Liu et al., 2019). This finding suggests that *Anaerostipes* may ethnically play a role in the development of AD. Furthermore, genus *Enterococcus*, a member of Lactobacillales, has been extremely studied as potential candidate probiotics by producing SCFAs for prevention of some human disease including irritable bowel syndrome symptoms (Avram-Hananel et al., 2010; Hanchi et al., 2018). Based on the results of this recent study and our findings, it would suggest that the reduction of SCFA-producing bacteria is involved in the development of the AD. To our knowledge, the protective roles of SCFAs against the formation of toxic soluble β-amyloid aggregates (Ho et al., 2018) and in regulating microglial inflammatory response *in vitro* (Huuskonen et al., 2004) involved in the development of AD are well documented. Thus, these results provide an insight that gut microbiota alterations may contribute to or exacerbate AD pathology through modulation of host metabolism.

To assess associations of genetic risk loci with a number of bacterial taxa for AD, we initially employed a considerably strict statistical cutoff (*q* < 0.05 in the MaAsLin) to avoid false-positive results. To our knowledge, no such connections have been demonstrated to date in previous studies yet. In our present study, our finding has implications for an increase only in ε4 allele of *APOE* genotype among 12 genetic variants involving the risk of the development of AD associated with the growth of the phylum Proteobacteria and family Enterococcaceae, independent of the individual’s AD status. As mentioned previously, phylum Proteobacteria was more abundant in patient with AD as pathogens. Previous work noted in healthy humans and mice that *APOE* genotypes were significantly associated with the reduction of butyrate-producing bacteria that could promote health (Tran et al., 2019). Thus, expanding these previous hypotheses of the function of expressed apoE mentioned previously (Mahley, 1988; Herz and Beffert, 2000; Elliott et al., 2007), our findings add evidence that the ApoE genotype may have impact on the pathophysiology of AD through specific bacteria. However, the mechanism that shapes the gut microbiome by the ApoE genotype to promote AD pathogenesis will require a range of studies with knockout animal models to be fully understood.

Our study has several limitations. First, the samples with which we could evaluate this result were relatively small, but provided sufficient statistical power to detect relevant associations. Second, one single-center study may limit the application of the genetic and microbial interactions. However, subjects are ethnically and geographically homogenous individuals to minimize some confounders, such as the dietary factor. Future studies should be extended to the associations observed in multicenters of differing backgrounds. Third, rather than shotgun metagenomic sequencing of the entire DNA, we performed 16S rRNA gene sequencing that limited data interpretation at species level and in functional information, but substantially broadened our understanding of overall bacterial structure and abundance in relation to AD. Moreover, the specific design of the study falls short of discerning the sequential causal relationship, which should be investigated in knockout animal models or in longitudinal studies before and after the onset of AD. Despite these limitations in this study, the results of AD-associated genetics and bacteria we found are in agreement with previous findings.

## Conclusion

Our study demonstrated that Proteobacteria significantly increased in patients with AD status and had a relationship with genetic risk APOE genotype. This study provided an insight that changes in the gut microbiota were associated with specific host genetic variants, which is important for understanding the AD pathogenesis. Moreover, it is proposed that targeting to modulate gut microbiota, especially the improvement of SCFA-producing bacteria and reduction of pathogens, might be an effective therapeutic strategy for disorders susceptible to gene host genetics.

## Data Availability Statement

The datasets presented in this study can be found in online repositories. The names of the repository/repositories and accession number(s) can be found in the article/Supplementary Material.

## Ethics Statement

The studies involving human participants were reviewed and approved by the Ethics Committee of School of Public Health at Shanghai Jiao Tong University for Human Subject Research. The patients/participants provided their written informed consent to participate in this study.

## Author Contributions

MH, GX, and HW designed and conceptualized the study, and revised the manuscript. MH, MR, and WL contributed to data collection and database generation. MH and GX performed the experiments, data analysis, and interpretation. MH drafted and revised the manuscript. All authors read and approved the manuscript.

## Conflict of Interest

The authors declare that the research was conducted in the absence of any commercial or financial relationships that could be construed as a potential conflict of interest.

## Footnotes

1 http://drive5.com/uparse/

2 https://preventivemedicine.usc.edu/download-quanto

## Supplementary Material

Supplementary Figure 1 Individual PCR fragments.

Supplementary Table 1 Primer information.
