# Supplementary figures and images for: APOE-ε4 Carrier Status and Gut Microbiota Dysbiosis in Patients With Alzheimer Disease
Source: Front Neurosci. 2021 Feb 24;15:619051. doi: 10.3389/fnins.2021.619051 (PMC7959830; doi:10.3389/fnins.2021.619051)

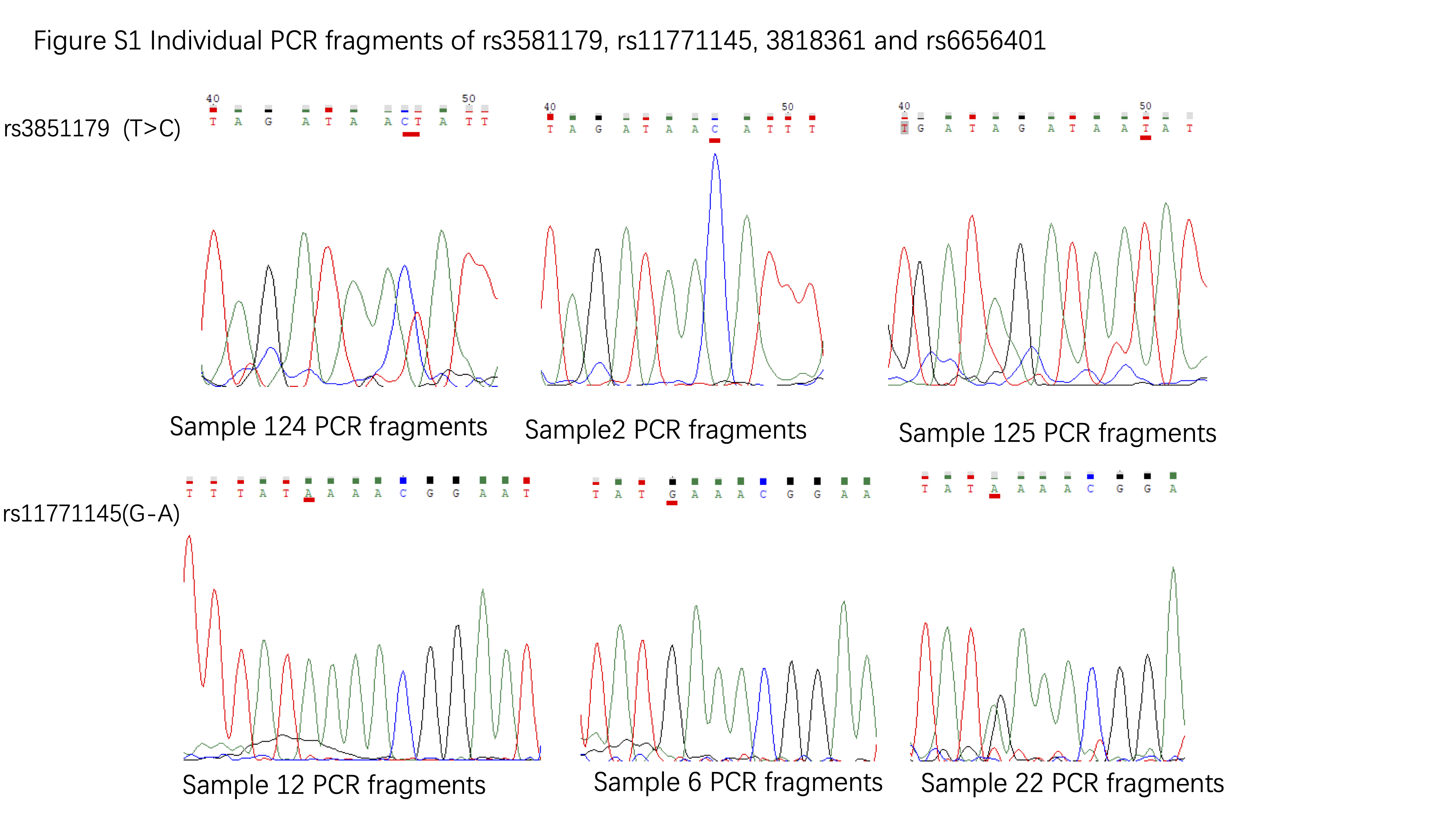

Supplement: Supplementary Figure 1 — Individual PCR fragments. [file Image_1.TIFF]

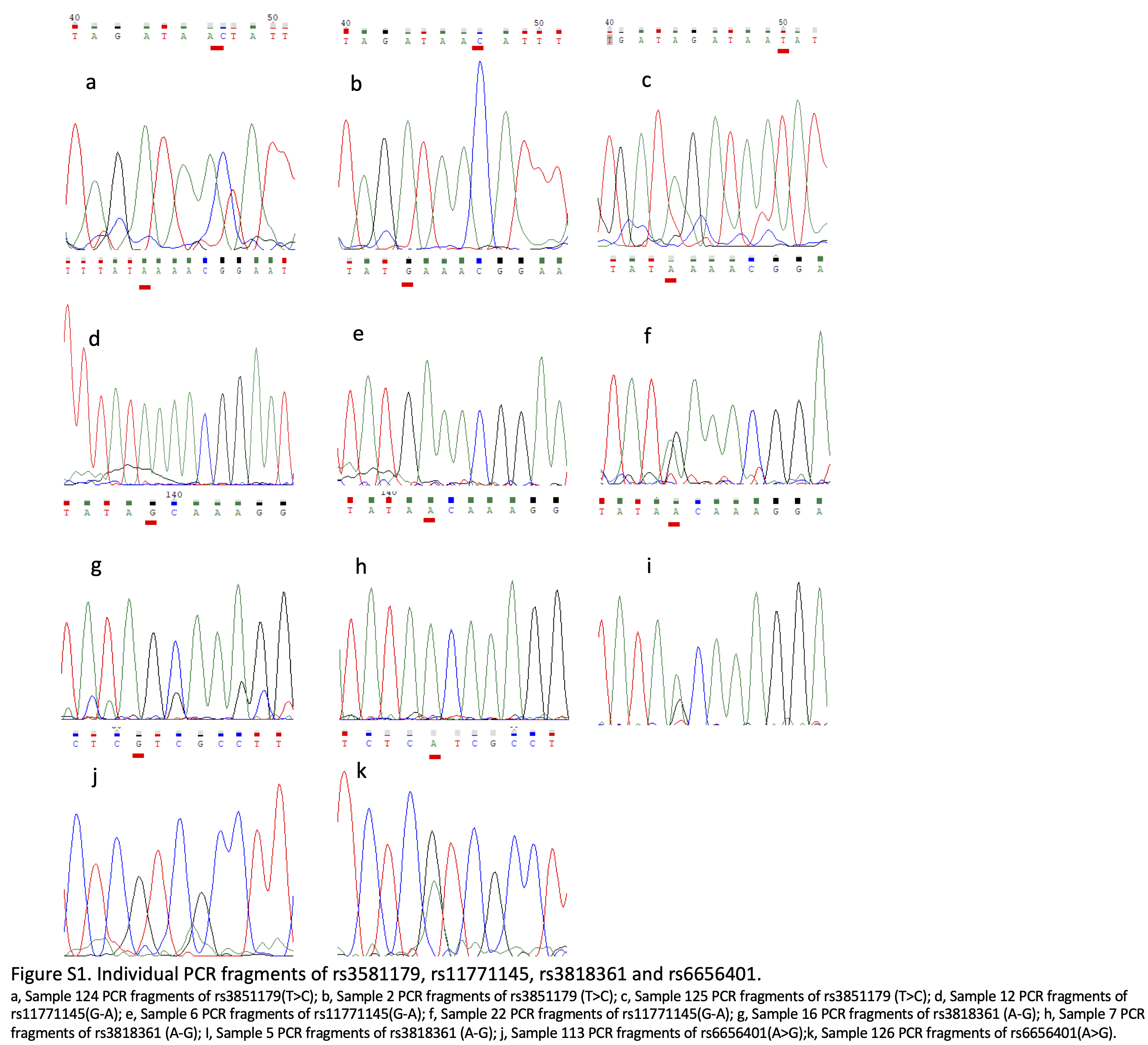

Supplement: Supplementary file 2 [file Image_2.TIFF]
