# Supplementary material for: APOE-ε4 Carrier Status and Gut Microbiota Dysbiosis in Patients With Alzheimer Disease
Source: Front Neurosci. 2021 Feb 24;15:619051. doi: 10.3389/fnins.2021.619051 (PMC7959830; doi:10.3389/fnins.2021.619051)
Supplement: Supplementary file 5 [file Table_3.docx]

**Faecal DNA extraction**

DNA was extracted from 200 mg of faeces using the E.Z.N.A Stool Extraction Kit following the manufacturer’s protocol (Omega Bio-tek, Norcross, GA, U.S.), and quantified using Nanodrop spectrophotometer (Thermo Scientific, Wilmington, DE). The barcoded primers 338F(5’-ACTCCTACGGGAGGCAGCAG-3’) and 806R (5’-GGACTACHVGGGTWTCTAAT-3’), targeting the V3-V4 variable regions of the 16S rRNA gene, were employed. Amplification reaction (initial activation step at 94 ̊C for 1 min followed by 30 cycles of 94 ̊C for 15s, 43 ̊C for 15s and 68 ̊C for 45s plus final incubation at 68 ̊C for 1 min) was performed in a total volume of 20μL containing 1 × FastPfu Buffer, 0.4μL of TransStartFastPfu DNA Polymerase (TransGen, Beijing, China), 0.8μL of each barcoded primer (5μM), 2μL of 2.5mM dNTPs, the concentration-adjusted DNA sample (10 ng). The amplifications of each sample were in triplicates. All PCR reactions were run on 2% agarose gels to verify correct amplification and were purified using an AxyPrep DNA Gel Extraction Kit (Axygen Biosciences, Union City, CA, USA). The purified PCR products were then quantified using the QuantiFluor-ST Real-time PCR System (Promega, USA) following the manufacturer’s protocol. An equal mass of amplicons from each sample were pooled and sequenced on the Illumina 300 Platform (Illumina, San Diego, USA).

**V3-V4 16S sequencing using Illumina Miseq 2×300bp**

Illumina MiSeq system was used to generate nucleotide-sequencing data for 77 samples with 436 of sequencing average length. Sequencing read pre-processing, including merging and demultiplexing was done. Forward and reverse sequencing reads were merged based on overlap to generate single reads using Flash 1.2.11 with the following criteria: (1) Reads were truncated at any site receiving an average quality score of <20 over a 50bp sliding window. (2) Sequences that overlapped > 10bp were merged according to their overlap with a mismatch < 2bp. (3) The sequences of each sample were separated according to barcodes (exactly matching) and primers (allowing two nucleotide mismatches), and reads containing ambiguous bases were removed. Raw sequence reads were quality trimmed using Trimmomatic software. The reads were then labelled according to 20-base barcodes located at the 3′ end of the read. Finally, the labelled reads were demultiplexed into separate FASTq files by sample ID.

**Operational taxonomic units picking and filtering**

The sequences were then clustered into operational taxonomic units (OTUs) using UPARSE (version 7.1 http://drive5.com/uparse/) with a novel “greedy” algorithm with a 97% of similarity and taxonomically classified using the Greengenes reference database [37]. Samples with less than 10 000 counts were removed. OTUs that were not present in at least 1% of our samples or with a low abundance (<0.01% of the total counts) were filtered out. The taxonomy of each 16S rRNA gene sequence was analyzed using the RDP Classifier algorithm (http://rdp.cme.msu.edu/) against the Silva (SSU128) 16S rRNA database using a confidence threshold of 70% [38]. The most abundant sequence within each OUT was used to represent the OTU. OTUs representing identical taxonomies were aggregated, and higher taxon levels were added when multiple OTUs represented that taxon. Due to the limitations of the resolution on taxonomical classification using 16S gene sequencing, we restricted our analysis to the genus level and above.
